# Differing Virulence of Healthy Skin Commensals in Mouse Models of Infection

**DOI:** 10.3389/fcimb.2018.00451

**Published:** 2019-01-21

**Authors:** Ian A. Myles, Ian N. Moore, Carlo R. Castillo, Sandip K. Datta

**Affiliations:** ^1^Laboratory of Clinical Immunology and Microbiology, National Institute of Allergy and Infectious Diseases, National Institutes of Health, Bethesda, MD, United States; ^2^Comparative Medicine Branch, National Institute of Allergy and Infectious Diseases, National Institutes of Health, Rockville, MD, United States

**Keywords:** microbiome, *Roseomonas mucosa*, *Staphylococcus*, biotherapeutic, atopic dermatis

## Abstract

**Introduction:** As therapies for atopic dermatitis (AD) based on live biotherapeutic products (LBP) are developed, the potential displacement of biotherapeutic strains, and species to mucosal sites where they are not naturally found is of investigative interest. However, formal assessment of the toxicity potential of healthy skin commensal organisms has not been reported in the literature. Our previous research indicates that topical application of live *Roseomonas mucosa* to treat AD was associated with clinical benefit on the skin, but the effects of exposure via inhalation, eye inoculation, and ingestion were unknown.

**Methods:** Herein we report our findings from mice inoculated with commensal strains of *R. mucosa*, coagulase negative *Staphylococci* (CNS), and *Pseudomonas aeruginosa*. Bacterial isolates were collected under clinical trial NCT03018275, however these results do not represent an interventional clinical trial.

**Results:** Our tested R. mucosa isolates did not display significant infection or inflammation. However, neutropenic mice inoculated with CNS had infection without major inflammation in pulmonary models. In contrast, systemic infection generated hepatic and splenic pathology for *P. aeruginosa* and CNS, which was worsened by the presence of neutropenia.

**Discussion:** Our results suggest that LBP derived from bacteria without significant infectivity histories, such as *R. mucosa*, may represent safer options than known pathobionts like *P. aeruginosa* and *Staphylococcus* spp. Overall, these results suggest that topically applied LBP from select skin commensals are likely to present safe therapeutic options and reinforce our prior clinical findings.

## Introduction

Live biotherapeutic products (LBP) are defined by the US Food and Drug Administration as any drug (other than vaccines) containing alive organisms that is intended to prevent, treat, or cure a human disease (FDA, 2016). Many LBP aim to alleviate disease through either direct or indirect manipulation of the microbiome (Olle, 2013). Several LBP are in development for the treatment of atopic dermatitis (AD) (Nakatsuji et al., 2017; Myles et al., 2018a), an allergic skin disease marked by barrier dysfunction, susceptibility to *Staphylococcus aureus* infection, immune dysregulation, and abnormalities of both the skin and gut microbiome (dysbiosis) (Grice and Segre, 2011; Kong et al., 2012; Eichenfield et al., 2014; Pyun, 2015; Chinthrajah et al., 2016; Petersen et al., 2018).

Our recent study demonstrated that isolates of *Roseomonas mucosa* harvested from healthy controls could reduce both subjective and objective symptoms of AD in adults and children (Myles et al., 2018a). Similar to our studies in mice (Myles et al., 2016a), topical application of live *R. mucosa* was associated with clinical benefit on the skin without signs of skin infection (Myles et al., 2018a). Furthermore, modeling possible systemic translocation by injection of *R. mucosa* intravenously (IV) into mice did not reveal any signs of infection or inflammation (Myles et al., 2018a). However, as LBP become more frequent, attention should be paid to the potential displacement of strains and species to mucosal sites where they are not naturally found. For example, the delivery device in our study (Myles et al., 2018a) produced an aerosolized solution which could allow the skin commensal to be inhaled or inoculated into the eye. Furthermore, treatment of pediatric patients raises the potential for inappropriate ingestion of skin bacteria into the gastrointestinal tract. To directly model these potential exposures, we inoculated mice with our treatment strains of *R. mucosa*. For comparison we evaluated a commensal strains of *Pseudomonas aeruginosa* and coagulase negative *Staphylococci* (CNS) (Myles et al., 2016a,b). *P. aeruginosa* was chosen because, while the specific strain used was a skin commensal from a healthy volunteer, the bacteria has established pathogenicity including bacteremia, as well as lung, eye, and skin infection (Morden and Berke, 2000; Juan et al., 2017). Furthermore, the strain of *P. aeruginosa* used in this study had similar beneficial impacts on our tissue models for AD—which may have prompted attempts at therapeutic use if the pathogenicity of *Pseudomonas* were not established. Strains of CNS were evaluated because of their current use in clinical trials for treatment of AD (Nakatsuji et al., 2017). Our CNS inoculum was a 1:1:1 mix of three previously described isolates *S. epidermidis, S. warneri*, and *S. hominis* (Myles et al., 2016b) speciated as previous described (Sastalla et al., 2017). Furthermore, since most reports of *R. mucosa* infection have been limited to patients with neutropenia and/or indwelling catheters (Han et al., 2003; Dé et al., 2004), CNS serves as a direct comparator due to the established pathology of CNS in these clinical settings (Meyers, 1986; von Eiff et al., 2001; Safdar and Maki, 2005).

In the current studies, mice were treated with either neutrophil-depleting antibodies or isotype control prior to exposure in systemic (intravenous; IV), ocular, gastric, or pulmonary (nasal route) models. Systemic infection generated hepatic and splenic pathology for select organisms. While our modeling could not recapitulate all possible immune deficient states, these results add to our prior clinical data to suggest that therapeutic use of normal skin commensals present safe therapeutic options when topically applied.

## Materials and Methods

### Patient Information

All human sample collection and processing were performed with approval of the National Institute of Allergy and Infectious Disease IRB, which approved the associated clinical trial (NCT03018275). All subjects gave written and verbal consent prior to sample collection. Participants ranged from 3 to 70+ years of age. Bacterial swabs were performed as previously described (Myles et al., 2016a,b).

### Isolate Selection

Three isolates of *R. mucosa* and one isolate of *P. aeruginosa* were selected based on previously described *in vitro* activity; the selected isolates inhibited the growth of *S. aureus* in broth culture and stimulated the vitamin D pathway in primary keratinocytes (Myles et al., 2016b). The three isolates of *R. mucosa* were also selected for use as a clinical therapeutic (Myles et al., 2018a). For Gram-positive organisms, three isolates of coagulase-negative *Staphylococci* and three isolates of *S. aureus* were selected. The three isolates of CNS were taken from the same individuals that provided the treatment strains of *R. mucosa* so that direct comparison of their commensal flora could be performed. For Gram-positive collection, swabs were plated on Mannitol Salt Agar plates. Colonies without evidence of mannitol fermentation were enumerated. A random sample of non-mannitol fermenting were tested for coagulase activity as well as 16S species confirmation was performed on all CNS isolates as previously described (Sastalla et al., 2017). *S. aureus* isolates were randomly selected from the collection of isolates from our cohort of patients with atopic dermatitis (Myles et al., 2016a). Both *Staphylococcus* spp (not shown) and *R. mucosa* isolates (Myles et al., 2016a) demonstrated the ability to colonize mice at least up to 14 days post-inoculation.

### Mice

Experiments were performed in both male and female mice that were age and sex matched within each experiment. C57BL/6 mice were purchased from Jackson Laboratories (Bar Harbor, ME). Intravenous injection with saline diluent or commensal organism was performed when mice were 7–8 weeks of age. Pulmonary models involved inoculating 10^6^ CFU of commensal organism in sterile saline in 10 mcL via intranasal route to anesthetized mice. Ocular exposure was performed by placing 10^6^ CFU of commensal organism in sterile saline in 5 mcL onto the eyes of anesthetized mice. Gavage was performed using a standard gavage needle (Fisherbrand 20G, Fisher Scientific, Waltham, MA) with 10^6^ CFU of commensal organism in 100 mcL of sterile saline. Weights were taken on days 0, 3, 6, and 7–10 for all challenges. On day 2-10, mice were sacrificed and the specified organs were harvested. Mice pre-treated with neutrophil-depleting antibody (anti-Gr1r; RB6-8C5; Invitrogen, Rockford, IL) were injected with 25mcg/mouse on day −1, 1, and 3 with day 0 representing infection. Isotype treatment was performed in an identical fashion using rat anti mouse IgG2b (RTK4530 Biolegend, San Diego, CA). Neutrophil depletion was verified similar to the previously described (Gaidamakova et al., 2012).

### Histology

Tissues were processed, sectioned, and stained with hematoxylin and eosin (H&E). All tissues were evaluated by a board-certified veterinary pathologist and photomicrographs were taken using an Olympus BX51 microscope and Olympus DP73 camera.

### Study Approval

Studies in humans were conducted under registered clinical trial NCT03018275 after approval from the NIAID institutional review board. All subjects were provided informed consent prior to their participation in the study. All murine experiments included in this study were carried out in accordance with the principles of the Basel Declaration and recommendations of NIAID Institutional Animal Care and Use Committee. The protocol was approved by the NIAID Institutional Animal Care and Use Committee.

## Results

### Select Commensal Organisms Induced Liver Pathology After Intravenous Injection

Our group has previously showed that IV injection with *R. mucosa* did not generate infection or illness in mice (Myles et al., 2016a). However, case reports suggest that neutropenia may be a necessary risk factor for *R. mucosa* infection (Dé et al., 2004). We injected a total of 10^6^ colony forming units (CFU) of a mixture of three strains of *R. mucosa* intravenously (IV) into mice after pre-treatment with either anti-Gr1 neutrophil depletion or isotype control. For comparison, separate mice were injected with 10^6^ CFU of a strain of *Pseudomonas aeruginosa* that was also harvested from a healthy control, or a mixture of three commensal strains of coagulase negative *Staphylococci* (CNS; total 10^6^ CFU) harvested from the same individuals as the *R. mucosa* isolates (Myles et al., 2016a,b) (Table 1). The dose of 10^6^ was selected because it is the current treatment regimen for the LBP under investigation for treatment of AD (Nakatsuji et al., 2017; Myles et al., 2018a). While any inadvertent inoculum would be expected to be lower than direct dosing, this study intended to model the maximal exposure from a single treatment (i.e., if the patient were to aspirate the entire dose).

**Table 1:** Summary of infections and results.

**Challenge route**	**Organ**	**Bacterial exposure**	**Pre-treatment**	**Pathology**	**Survival (%)**
IV	Liver	*R. mucosa*	Isotype	Rare foci of inflammation, sterile	100
IV	Liver	*R. mucosa*	Anti-Gr1	Rare foci of inflammation, bacterial presence in tissue	100
IV	Liver	*P. aeruginosa*	Isotype	Moderate numbers of large inflammatory foci, sterile	100
IV	Liver	*P. aeruginosa*	Anti-Gr1	Histologically normal, bacterial presence in tissue	0
IV	Liver	*Coag. Neg. Staph*	Isotype	Rare foci of inflammation, high bacterial tissue burden	100
IV	Liver	*Coag. Neg. Staph*	Anti-Gr1	Rare foci of inflammation, high bacterial tissue burden	25
IV	Spleen	*R. mucosa*	Isotype	WNL, sterile	100
IV	Spleen	*R. mucosa*	Anti-Gr1	Loss of follicular definition with expansion of B-cell zone, sterile	100
IV	Spleen	*P. aeruginosa*	Isotype	Loss of follicular definition with expansion of B-cell zone, sterile	100
IV	Spleen	*P. aeruginosa*	Anti-Gr1	Severe loss of follicular definition with expansion of B-cell zone, tangible body macrophages containing degenerative cellular debris; bacterial presence in tissue	0
IV	Spleen	*Coag. Neg. Staph*	Isotype	Loss of follicular definition with expansion of B-cell zone, high bacterial burden in tissue	100
IV	Spleen	*Coag. Neg. Staph*	Anti-Gr1	Loss of follicular definition with dramatic increase in the number of macrophages in the red pulp	25
IV	Kidney	*R. mucosa*	Isotype	WNL, sterile	100
IV	Kidney	*R. mucosa*	Anti-Gr1	WNL, sterile	100
IV	Kidney	*P. aeruginosa*	Isotype	Histologically normal, bacterial presence in tissue	100
IV	Kidney	*P. aeruginosa*	Anti-Gr1	Histologically normal, bacterial presence in tissue	0
IV	Kidney	*Coag. Neg. Staph*	Isotype	Histologically normal, high bacterial burden in tissue	100
IV	Liver	*Coag. Neg. Staph*	Anti-Gr1	Histologically normal, high bacterial burden in tissue	25
Pulmonary	Lung	All	All	WNL, sterile except for neutropenia with *Coag. Neg. Staph*.	100
Gavage	Stomach	All	All	WNL, sterile	100
Ocular	Eye	All	All	WNL, sterile	100

*Mice were challenged either via intravenous (IV), pulmonary, oral gavage, or ocular routes at 10^6^ colony forming units per exposure. Groups were treated with either isotype control or neutrophil depleting antibodies (anti-Gr1) prior and during infectious challenge as indicated. Results from histologic examination and bacterial burden enumeration from homogenized tissue are presented. Bacterial burden was assessed one day after infection. Survival at day 10 also presented for all groups. See Supplemental Figures 1, 2 for all histologic images. Data represents three independent experiments from male, female, C57BL/6, and Balb/cJ mice that were age, strain, and sex matched within each experiment (N = 4-5 mice per group, per experiment). WNL, within normal limits*.

**Figure 1:**
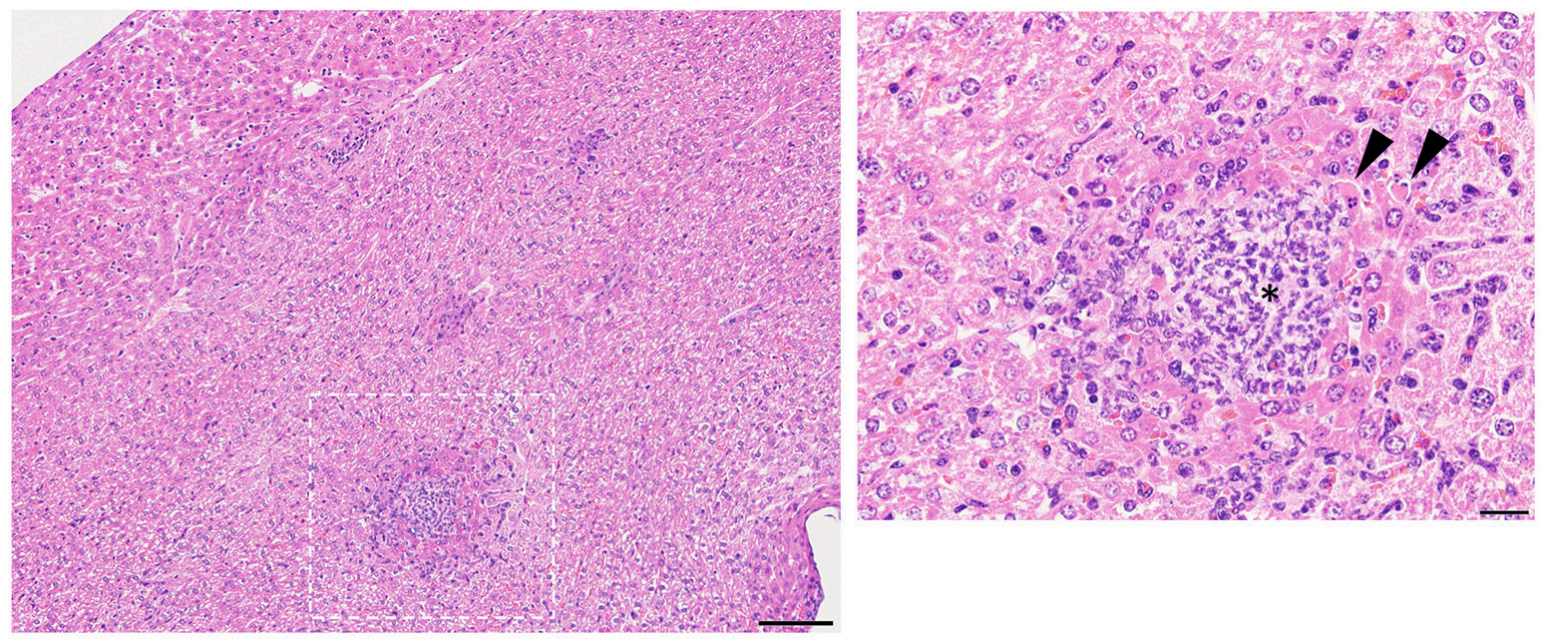
Representative liver pathology. Mice injected intravenously as in Table 1 after treatment with either isotype control or neutrophil depleting antibodies (anti-Gr1) as indicated. The liver was harvested 2–10 days after exposure. A representative image of the resultant pathology is shown on left with close up image of boxed area on right. Histologic examination revealed some sections of the liver contained multifocal areas of inflammation characterized by central collections of viable and degenerative neutrophils and macrophages (asterisk). These were often surrounded by degenerative and necrotic, hypereosinophilic hepatocytes (arrows). Size bars indicate 100 μm. Please see Supplemental Figure 1 for liver images from all groups. Data represents three independent experiments from male, female, C57BL/6, and Balb/cJ mice that were age, strain, and sex matched within each experiment (*N* = 4–5 mice per group, per experiment).

**Figure 2:**
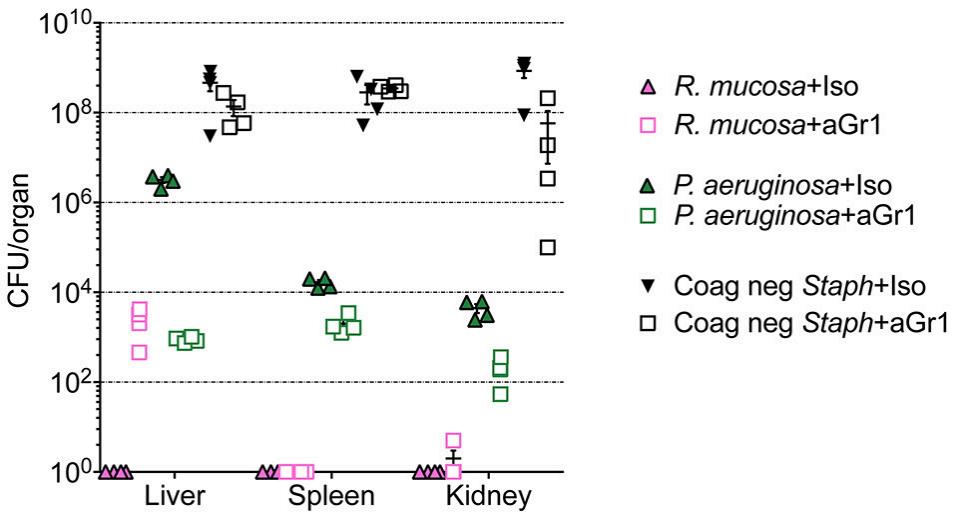
Bacterial enumeration from homogenized organs. Mice injected intravenously as in Table 1 after treatment with either isotype control (Iso; filled triangles) or neutrophil depleting antibodies (anti-Gr1; open boxes) as indicated. The liver, spleen, and kidneys were harvested 2–10 days after exposure, homogenized, and plated on bacterial growth agar after serial dilutions. Twenty-four to Seventy-two hour later, colony forming units (CFU) were enumerated. Data represents three independent experiments from male, female, C57BL/6, and Balb/cJ mice that were age, strain, and sex matched within each experiment. *N* = 4–5 mice per group, per experiment.

Mice were monitored for up to 10 days after infection by disinterested animal technicians in a blinded fashion. Similar to our prior studies (Myles et al., 2016a), mice treated with isotype antibody and injected with *R. mucosa* displayed no histologic abnormality in the spleen or kidney after IV inoculation, while liver histology demonstrated only rare foci of inflammation (Table 1; Figure 1; Supplemental Figure 1). Homogenized organs did not grow any bacteria, suggesting that *R. mucosa* could not proliferate in the systemic compartment and/or was rapidly cleared (Figure 2).

Histologic examination in neutropenic mice inoculated with IV *R. mucosa* revealed mild-moderately severe, multifocal, random, hepatic inflammation consisting of macrophages, and some neutrophils in the liver; rare areas of extramedullary hematopoiesis as indicated by an increase in erythroid precursor cells were also present (Table 1; Supplemental Figure 1). There was no evidence of pathology observed in the sections of spleen or kidney (Table 1; Supplemental Figure 1). However, tissue infection was present in liver and, to a lesser extent, kidney for the neutropenic mice (Figure 2).

**Figure 3:**
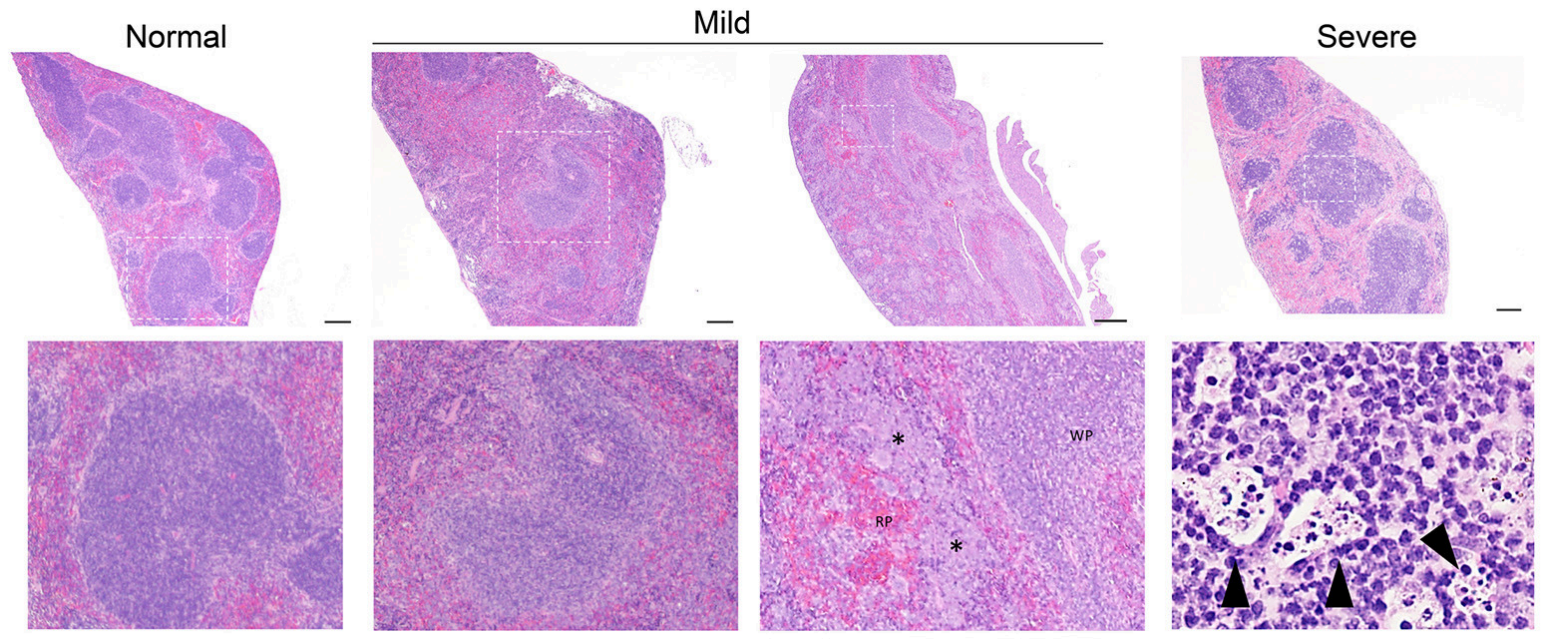
Representative splenic pathology. Mice injected intravenously as in Table 1 after treatment with either isotype control or neutrophil depleting antibodies (anti-Gr1) as indicated. The spleen was harvested 2–10 days after exposure. A representative image of normal (*R mucosa* exposed), mild pathology (Coagulase negative *Staphylococcus*; CNS exposed), or severe pathology (*P. aeruginosa* infected neutropenic mice) is shown (see Table 1). Mild pathology for Gram-negative exposures was marked by alteration of follicular definition with expansion of B-cell zone (hyperplasia). For neutropenic mice injected with CNS, within the “red pulp” (RP; vascular spaces) and adjacent to the “white pulp” (WP; lymphoid tissue) there were dense focal and coalescing clusters of histiocytic cells (asterisk); these changes can be seen throughout the entire splenic section. Severe pathology after *P. aeruginosa* injection was marked by severe alteration of follicular definition with expansion of B-cell zone (hyperplasia) as well as tingible body macrophages containing degenerate cellular debris. Upper row images taken at 10x with expansion of boxed areas shown below. Size bars indicate 100 μm. Please see Supplemental Figure 1 for liver images from all groups. Data represents three independent experiments from male, female, C57BL/6, and Balb/cJ mice that were age, strain, and sex matched within each experiment (*N* = 4-5 mice per group, per experiment).

Isotype-treated mice injected with a commensal strain of *P. aeruginosa* revealed pronounced hepatic changes; regions of inflammation were more extensive, slightly more numerous, and were associated with occasional areas of hepatic necrosis (Table 1; Figure 1; Supplemental Figure 1). Splenic histology in mice injected with *P. aeruginosa* was also consistent with infection and demonstrated changes in normal splenic architecture that was largely attributable to lymphoid hyperplasia (Figure 3; Supplemental Figure 1) and bacterial growth was present in homogenized spleens (Figure 2).

While hepatic histology in neutropenic mice injected with IV *P. aeruginosa* was normal (Table 1; Supplemental Figure 1), bacteria were present in the tissue (Figure 2). Splenic histology of neutropenic mice injected with *P. aeruginosa* showed pronounced inflammatory changes as indicated by some destruction of lymphoid tissue and a marked increase in the number of tingible body macrophages (Figure 3). These are macrophages that often contain multiple small vacuoles, mostly within the lymphoid follicles, which further contain abundant pyknotic cellular debris (Figure 3; Supplemental Figure 1). Splenic tissue also demonstrated bacterial seeding in neutropenic mice injected with *P. aeruginosa* (Figure 2). Furthermore, injection with *P. aeruginosa* was 100% lethal in neutropenic mice (Table 1).

While neutropenia is a risk factor of staphylococcal infection (Dean, 2010; Dryden, 2010), low or dysfunctional neutrophils are not a requirement for staphylococcal virulence (Myles and Datta, 2012; Natsis and Cohen, 2018). Consistent with this observation, liver pathology in mice injected with CNS demonstrated rare foci of inflammation (Table 1; Supplemental Figure 1) but showed high bacterial tissue burden (Figure 2). Splenic tissue demonstrated a loss of follicular definition with expansion of B-cell zone (Table 1; Figure 3; Supplemental Figure 1) with a high bacterial burden in tissue (Figure 2). Renal tissue was found to be within normal histologic limits (Supplemental Figure 1); however, high bacterial counts were also extracted from the kidney (Figure 2). Neutropenic mice injected with CNS also demonstrated loss of splenic architecture characterized by densely cellular, clustered, predominantly macrophage-rich (granulomatous) cellular infiltration in many regions of the red pulp (Table 1; Figure 3; Supplemental Figure 1). Pathology of the kidney and liver were similar to isotype treated mice; however, survival was only 25% in neutropenic mice injected with CNS (Table 1).

While this model does not recapitulate the indwelling catheters or potential immune suppressant effects unique to blood cancers, these data indicate that commensal strains of CNS and *P. aeruginosa* may pose a risk after systemic exposure, whereas neutropenia may be a necessary risk factor for *R. mucosa* pathology.

### Tested Commensal Organisms Failed to Generate Inflammatory Changes in Epithelial Challenge Models

**Figure 4:**
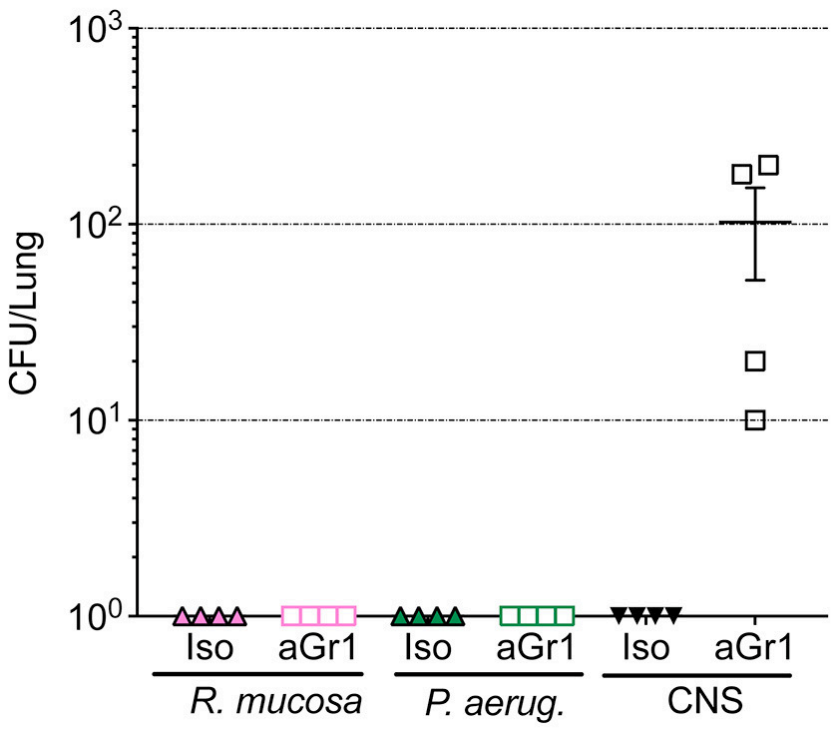
Bacterial enumeration from homogenized lungs. Mice were inoculated via nasal passage in Table 1 after treatment with either isotype control (Iso; filled triangles) or neutrophil depleting antibodies (anti-Gr1; open boxes) as indicated. The lungs were harvested 2–10 days after exposure, homogenized, and plated on bacterial growth agar after serial dilutions. 24–72 h later, colony forming units (CFU) were enumerated. Data represents three independent experiments from male, female, C57BL/6, and Balb/cJ mice that were age, strain, and sex matched within each experiment. *N* = 4-5 mice per group, per experiment.

Aerosolized bacteria could present the potential for inadvertent inhalation. To assess this directly, nasal inoculation was performed on mice using 10^6^ CFU of *R. mucosa, P. aeruginosa*, or CNS. Seven days after inoculation there were no histologic signs of inflammation or infection for any commensal challenge in any isotype-treated or neutropenic mouse (Table 1; Supplemental Figure 1). Viable bacterial colonies were only detectable in the pulmonary tissue of neutropenic mice inoculated with CNS (Figure 4). In contrast, known pathogens such as the methicillin resistant *S. aureus* (MRSA) isolate USA300-LAC have demonstrated marked pathology in pulmonary and systemic models in wild type mice (Gaidamakova et al., 2012; Myles et al., 2018b).

A single case report has described an eye infection with *R. mucosa* (Bhende et al., 2017). To partially model this report as well as potential ocular exposure during aerosolized treatment, we inoculated 10^6^ of *R. mucosa, P. aeruginosa*, or CNS into the eye of isotype-treated and neutropenic mice. Similar to the pulmonary challenge, histology of the eyes 7 days after inoculation was within normal limits for all mice (Table 1; Supplemental Figure 2). Consistent with these histologic findings, homogenized tissues plated on bacterial culture plates were negative for any bacterial growth (not shown). Lastly, to model possible improper oral administration, mice were gavaged with our commensal organisms. Histologic evaluation of the stomachs 2–10 days after challenge were normal for all mice, including neutropenic mice (Table 1; Supplemental Figure 2). These data suggest that for the selected isolates, exposure of epithelial and mucosal surfaces is unlikely to present a risk for infectious or inflammatory complications.

### Tested Commensal Organisms Were Present On Humans in Low Abundance

**Figure 5:**
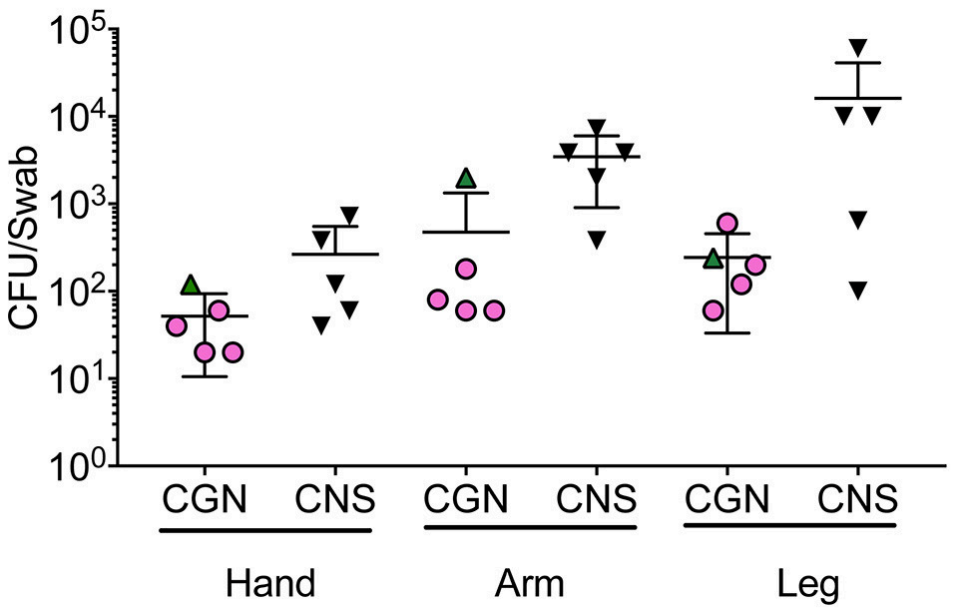
Bacterial burden on human skin for selected isolates. **Five** health controls were swabbed for Gram negative and Gram positive growth. Swabs were initially moistened before rubbing on the hand, antecubital fossa (arm), or popliteal fossa (leg) for 30 s. Swabs were then placed in 2 mL of either R2A (Culturable Gram-negatives; CGN) or Brain Heart Infusion (Gram positive) broth and vortexed for 30 s before 100 mcL was plated onto culture agar (R2A for Gram negative organisms, mannitol salt agar for Gram positive). For Gram-positive bacteria, colony forming units (CFU) of the non-mannitol fermenting colonies were enumerated 24 h after plating and multiplied by 20 to represent the total for the 2 mL culture volume. For Gram-negative, CFU on R2A plates were enumerated 72 h after plating and also multiplied by 20 to represent the 2 mL culture volume. Subsequent to collection, coagulase positivity and 16S identification were performed to confer CNS designation.

To assess how natural species translocation compared to the CFU burden used in this study and current treatment protocols (Nakatsuji et al., 2017; Myles et al., 2018a), we cultured the hands of healthy controls to enumerate the bacterial burden per square inch to assess potential passive transfer from such behaviors as rubbing one's eye. We found that hand burden of Gram-negative organisms ranged from 20–120 CFU per square inch (in^2^), while CNS carriage was 40–720 CFU/in^2^ (Figure 5). While colonization burden on the arms and legs were higher than the hands (Figure 5), rates were still far lower than current treatment protocols (Nakatsuji et al., 2017; Myles et al., 2018a). This suggests that the safety of therapeutics containing topical commensals may not be automatically assumed if treatment doses are far greater than natural exposure.

## Discussion

Numerous direct and indirect microbiome-targeting therapies are currently under investigation (Olle, 2013). Moving forward, it may be important to establish expectations for evaluating the impacts of relocation of commensals from one body site to another prior to human exposure. While some movement of bacterial isolates may be expected from behaviors such as rubbing the eye or fecal-oral contamination, the effects of species translocation cannot be assumed given that microbial therapies may represent much higher inoculum than natural exposure. For example, our culture techniques demonstrated that the normal human hand carries far fewer CFU of the selected commensals than the current AD treatment protocols (Nakatsuji et al., 2017; Myles et al., 2018a). Therefore, researchers may not be able to assume that a given commensal will remain mutualistic after being concentrated by several orders of magnitude prior to clinical use.

Treatment with AD has also been attempted using strains of *Staphylococcus epidermidis* that produce lantibiotics that synergize with host anti-microbial peptides for inhibition of *S. aureus* growth (Nakatsuji et al., 2017). While the specific strains of CNS used by other groups were not available for our experiments, we collected CNS isolates from the same individuals who were the source of our tested strains of *R. mucosa* (Myles et al., 2016a,b). These isolates provide unique comparisons given the *R. mucosa* from the same individuals failed to generate pathology in either mouse models or human trials. In contrast with *R. mucosa*, systemic inoculation of our CNS strains generated hepatic and splenic inflammatory changes even in the absence of neutropenia. Therefore, our results suggest that LBP derived from bacteria without significant infectivity histories, such as *R. mucosa*, may represent safer options than known pathobionts like *P. aeruginosa* and *Staphylococcus* spp.

Our neutropenic model does not directly assess the potential effect of unique immune defects that may be associated with indwelling catheters, diabetes, or specific cancers. Another significant limitation of our study is the non-traditional approach to toxicity assessment. Under traditional pharmaceutical design, animal and/or tissue models are exposed to increasing doses of the novel drug until toxicity is seen. The dosage immediately below the one that generates signs of toxicity is the “no adverse event level” (NOAEL). The subsequent human trials designed to discover the ideal therapeutic dose use the NOAEL as a guide for the upper limit of exposure.

Our current study takes the opposite approach by applying the pre-derived therapeutic human dose and assessing for toxicity in mice. Indeed, the more traditional NOAEL derivation may be indicated in LBP assessment if application devices allow for microbial growth to higher inoculums (e.g., expansion of a gut microbe in a yogurt-based delivery system). However, the traditional approach to NOAEL derivation through exposing mice to increasing dosages until toxicity is seen may not be valid for LBP. Quorum-sensing mechanisms (Hentzer and Givskov, 2003; Yarwood and Schlievert, 2003) as well as progression to stationary phase growth (Myles et al., 2016b) place a real-world limit on the concentration of bacterial products in ways that do not exist for traditional medications. A reasonable regulatory approach may be to assess the virulence of future LBP up to the highest CFU concentration achievable in the product delivery device and/or packaging. As far as these factors pertain to *R. mucosa*, our previous work assessed intravenous (IV) exposure 100-fold greater than our treatment dose and our application device does not allow for bacterial proliferation while in storage (Myles et al., 2018a). In addition, while regulatory bodies have not determined the need for NOAEL derivation for LBP (FDA, 2016), our study still provides novel insights into the safety of LBP by providing the first toxicity assessment of an LBP as well as making novel comparisons of virulence between commensal species.

Our findings in mouse models, combined with our human safety data (Myles et al., 2018a), suggests that treatment with *R. mucosa* is unlikely to cause direct epithelial pathology up to the doses tested. Additional assessment of the global impact on the microbiome with LBP use may also be warranted to evaluate the potential for inducing what might be thought of as “iatrogenic dysbiosis.” However, for select organisms with pathobiont potential, immediate caution is warranted to prevent against systemic spread and to prevent exposure to patients with neutropenic disorders. While systemic translocation is highly unlikely from a topically applied live biotherapeutic, exclusion criteria for clinical trials may need to consider the exposure risk of both patients and co-habitants with immune defects or risk of systemic translocation.

## Author Contributions

IAM designed the studies and performed experiments, and wrote the manuscript. CC assisted in all experiments. INM provided pathology assessments for all histology slides and helped write the manuscript. SD oversaw the project. All authors critically reviewed the manuscript.

### Conflict of Interest Statement

The authors declare that the research was conducted in the absence of any commercial or financial relationships that could be construed as a potential conflict of interest.

## Supplementary Material

Supplemental Figure 1 Representative images from IV exposure and pulmonary tissue assessment. Mice injected intravenously or pulmonary route as in Table 1 were sacrificed 2–10 days after injection. Groups were treated with either isotype control or neutrophil depleting antibodies (anti-Gr1) prior and during infectious challenge as indicated. Histologic examination of liver, spleen, kidney, and lung was performed. A representative image from each group, for each organ is presented. Data represents three independent experiments from male, female, C57BL/6, and Balb/cJ mice that were age, strain, and sex matched within each experiment. *N* = 4-5 mice per group, per experiment, images presented are all from same experiment.

Supplemental Figure 2 Representative images from ocular and gastric exposure tissue assessment. Mice exposed via oral gavage or ocular routes as in Table 1 were sacrificed 2–10 days after exposure. Groups were treated with either isotype control or neutrophil depleting antibodies (anti-Gr1) prior and during infectious challenge as indicated. Histologic examination stomach and eyes was performed. A representative image from each group, for each organ is presented. Data represents two independent experiments from male, female, C57BL/6, and Balb/cJ mice that were age, strain, and sex matched within each experiment. *N* = 4-5 mice per group, per experiment, images presented are all from same experiment.
