# Supplementary figures and images for: Differing Virulence of Healthy Skin Commensals in Mouse Models of Infection
Source: Front Cell Infect Microbiol. 2019 Jan 21;8:451. doi: 10.3389/fcimb.2018.00451 (PMC6348709; doi:10.3389/fcimb.2018.00451)

Supplemental Figure 1

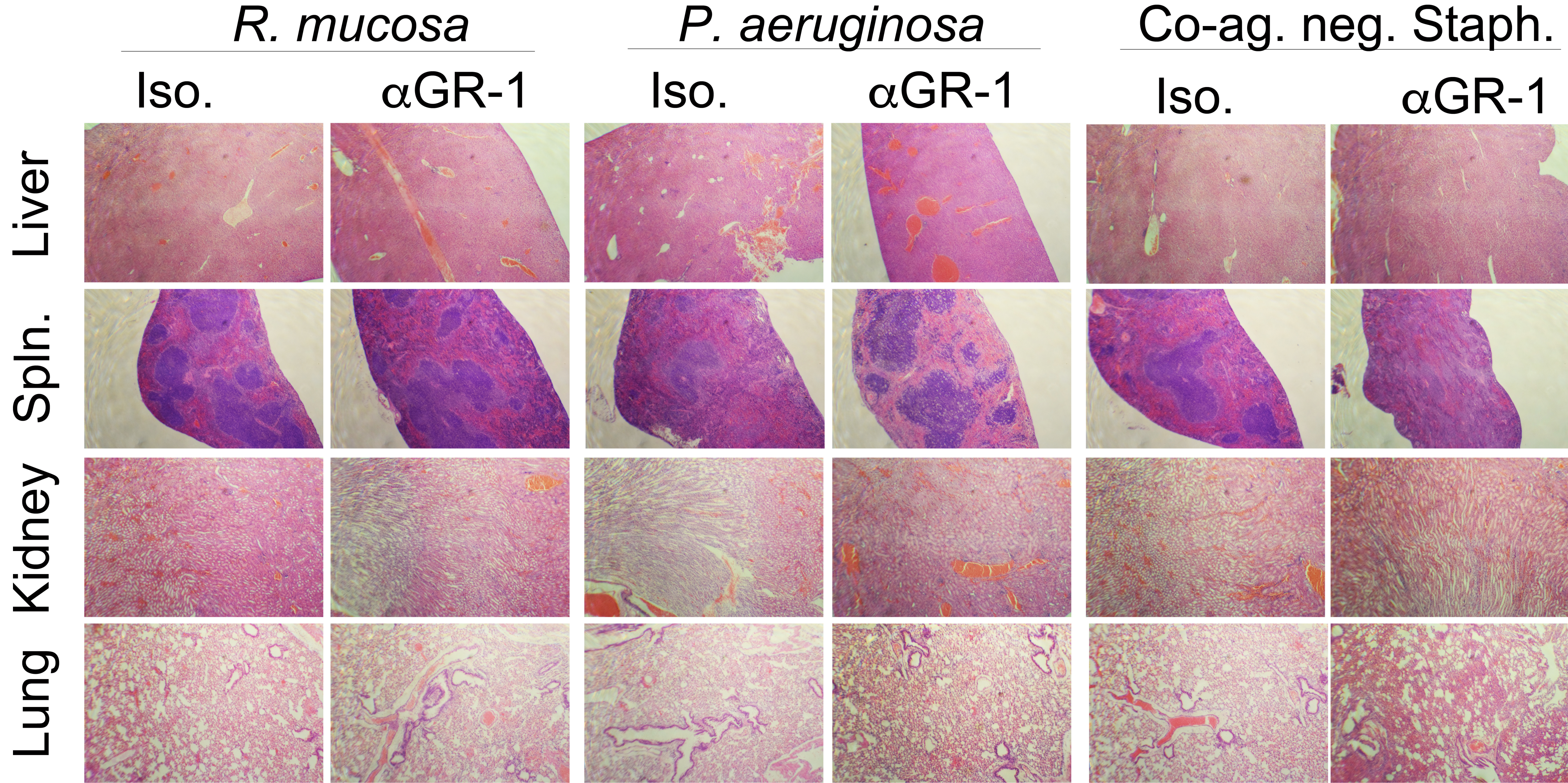

Supplement: Supplemental Figure 1 — Representative images from IV exposure and pulmonary tissue assessment. Mice injected intravenously or pulmonary route as in Table 1 were sacrificed 2–10 days after injection. Groups were treated with either isotype control or neutrophil depleting antibodies (anti-Gr1) prior and during infectious challenge as indicated. Histologic examination of liver, spleen, kidney, and lung was performed. A representative image from each group, for each organ is presented. Data represents three independent experiments from male, female, C57BL/6, and Balb/cJ mice that were age, strain, and sex matched within each experiment. N = 4-5 mice per group, per experiment, images presented are all from same experiment. [file Data_Sheet_1.PDF]

*R. mucosa*

*P. aeruginosa*

Co-ag neg. Staph

Eye

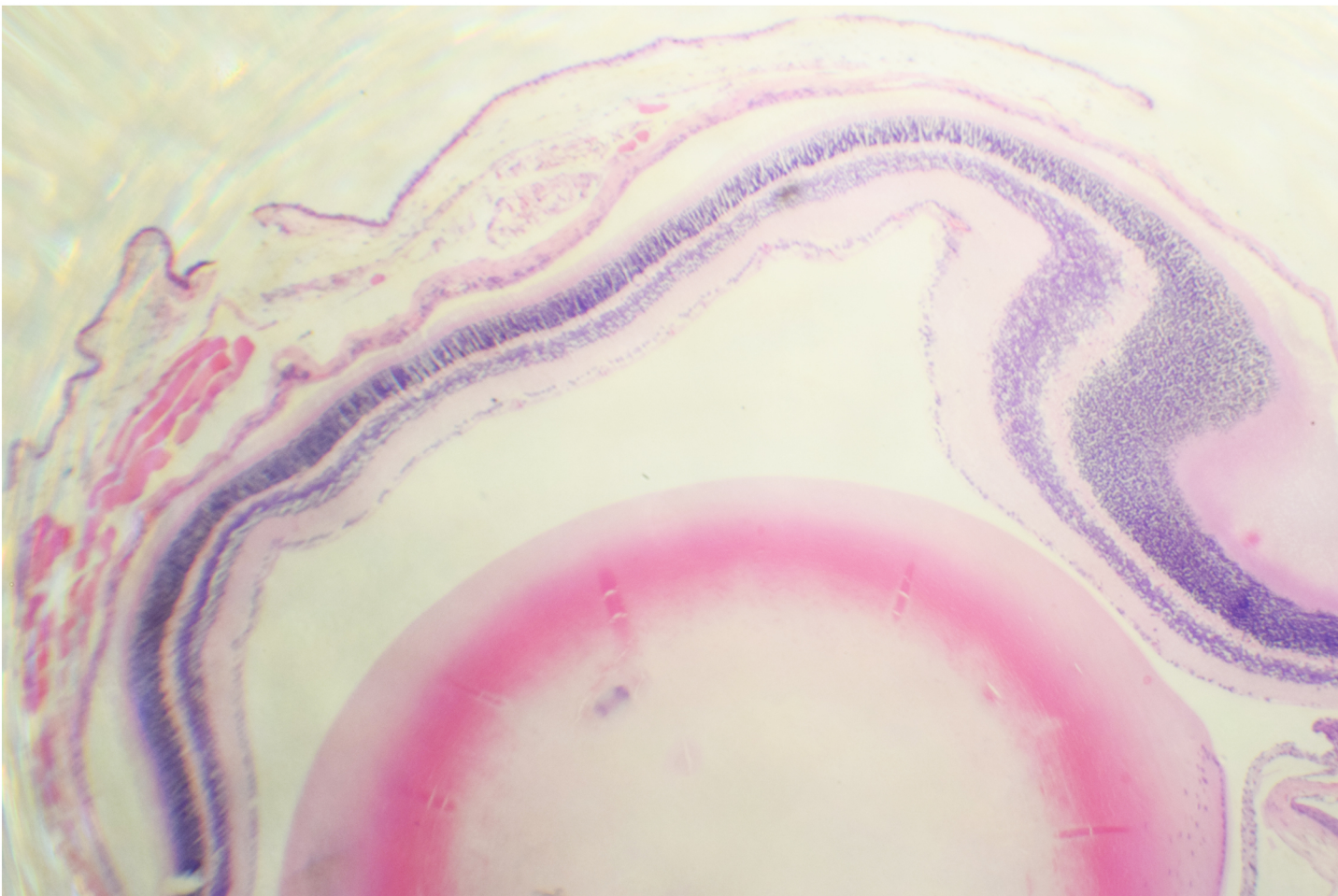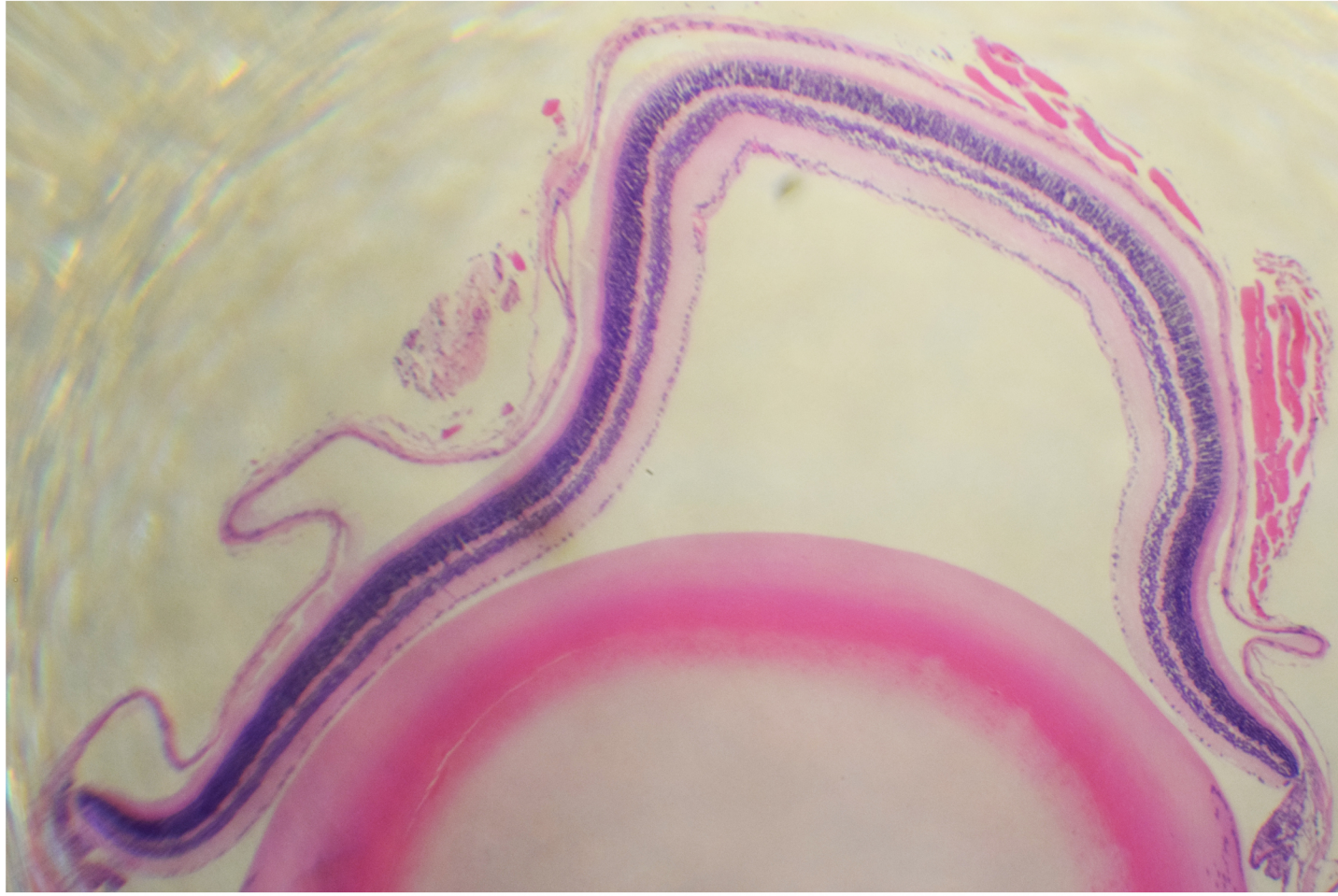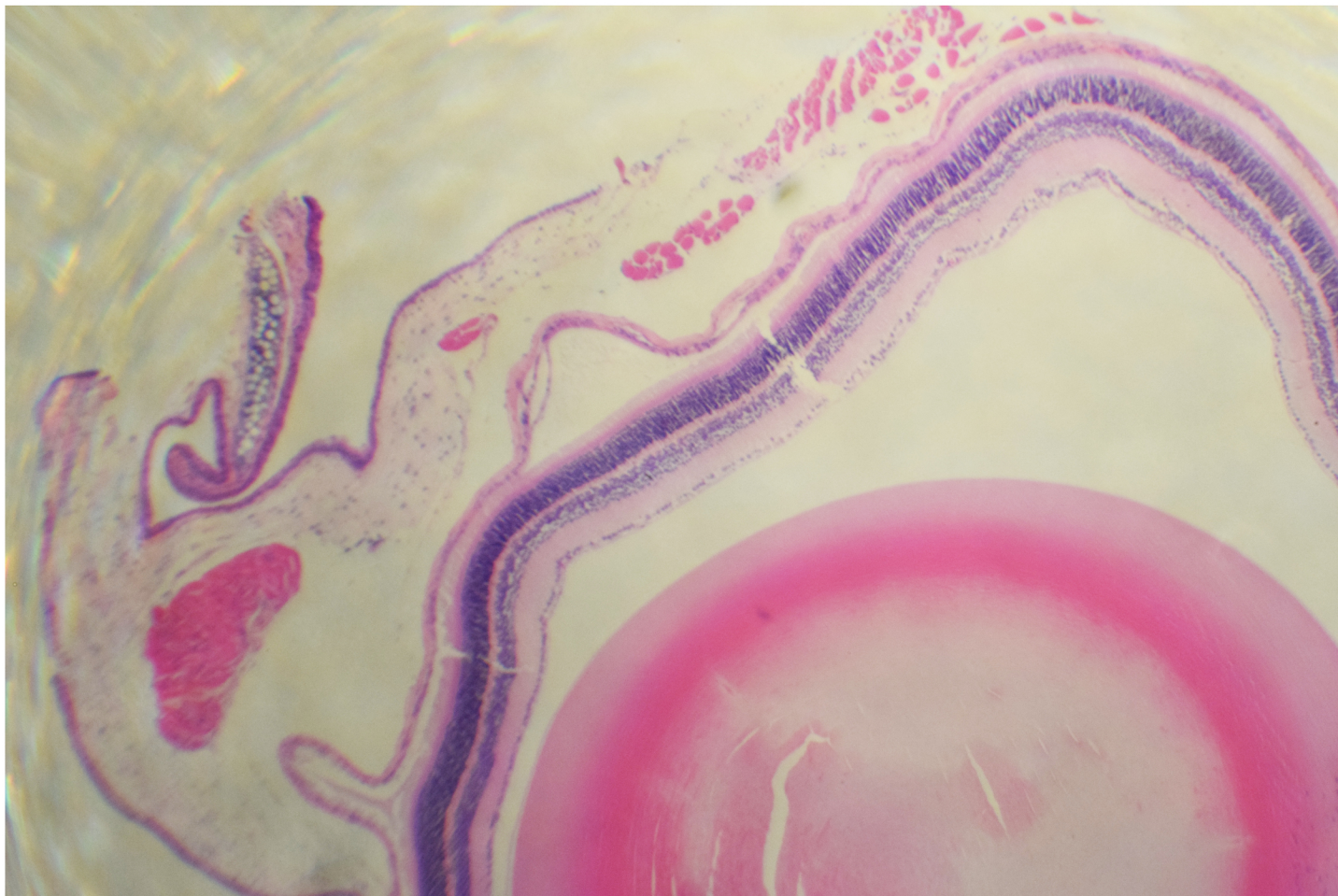

Stomach

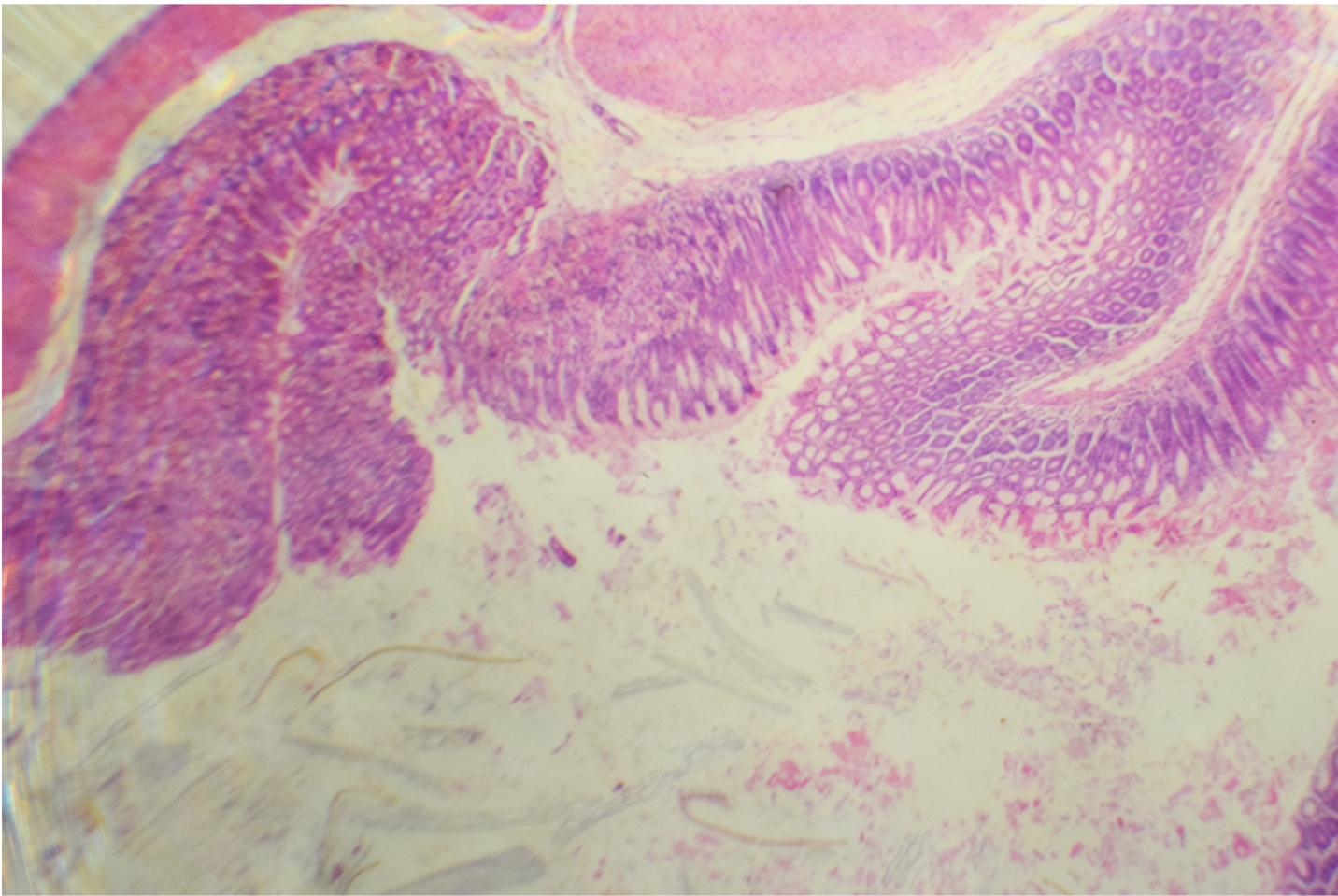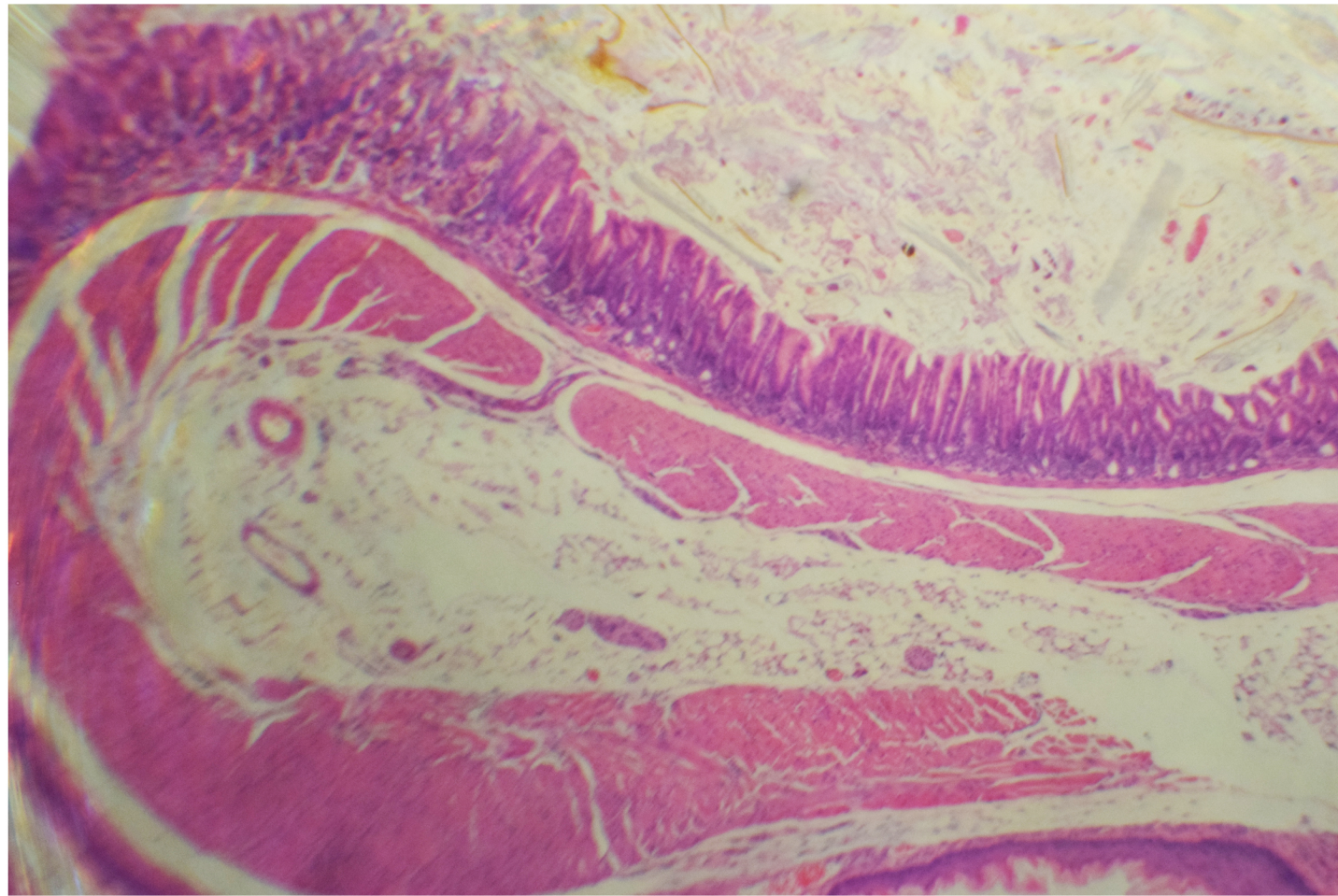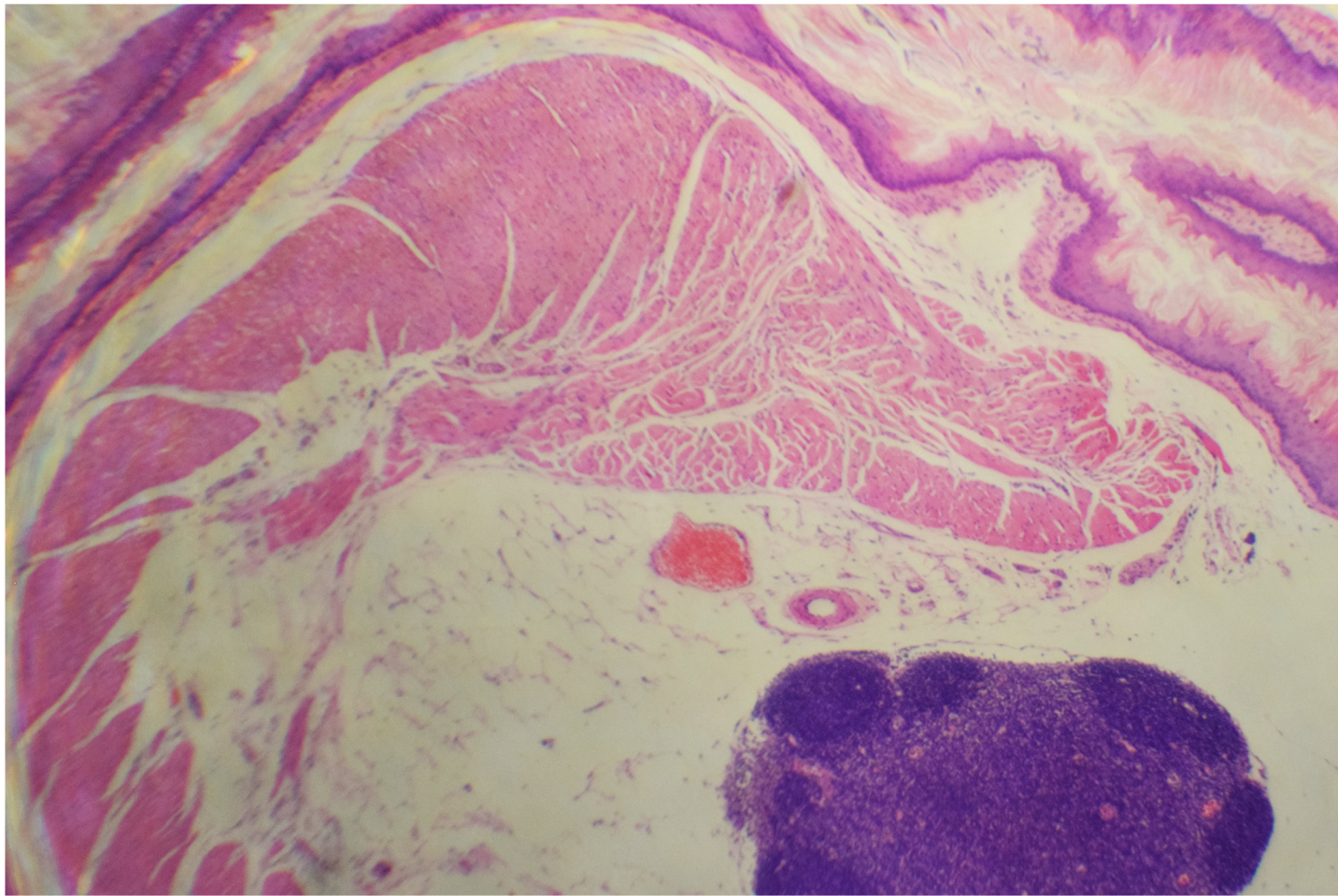

Supplement: Supplemental Figure 2 — Representative images from ocular and gastric exposure tissue assessment. Mice exposed via oral gavage or ocular routes as in Table 1 were sacrificed 2–10 days after exposure. Groups were treated with either isotype control or neutrophil depleting antibodies (anti-Gr1) prior and during infectious challenge as indicated. Histologic examination stomach and eyes was performed. A representative image from each group, for each organ is presented. Data represents two independent experiments from male, female, C57BL/6, and Balb/cJ mice that were age, strain, and sex matched within each experiment. N = 4-5 mice per group, per experiment, images presented are all from same experiment. [file Data_Sheet_2.PDF]
